# Detecting halfmetallic electronic structures of spintronic materials in a magnetic field

**DOI:** 10.1038/s41598-021-97992-z

**Published:** 2021-09-20

**Authors:** H. Fujiwara, R. Y. Umetsu, F. Kuroda, J. Miyawaki, T. Kashiuchi, K. Nishimoto, K. Nagai, A. Sekiyama, A. Irizawa, Y. Takeda, Y. Saitoh, T. Oguchi, Y. Harada, S. Suga

**Affiliations:** 1 Division of Materials Physics, Graduate School of Engineering Science, Osaka University, 1-3 Machikaneyama, Toyonaka, Osaka 560-8531 Japan; 2 Institute for Materials Research, Tohoku University, 2-1-1 Katahira, Sendai, Miyagi 980-8577 Japan; 3 Center for Spintronics Research Network, Tohoku University, 2-1-1 Katahira, Sendai, Miyagi 980-8577 Japan; 4 Center for Science and Innovation in Spintronics, 2-1-1 Katahira, Sendai, Miyagi 980-8577 Japan; 5 SANKEN, Osaka University, 8-1 Mihogaoka, Ibaraki, Osaka 567-0047 Japan; 6 Institute for Solid State Physics, The University of Tokyo, 5-1-5 Kashiwanoha, Kashiwa, Chiba 277-8581 Japan; 7 Synchrotron Radiation Research Organization, The University of Tokyo, 1-1-1 Koto, Sayo-cho, Sayo, Hyogo 679-5148 Japan; 8 Present Address: Institute for Advanced Synchrotron Light Source, National Institutes for Quantum and Radiological Science and Technology, 6-6-11 Aoba, Sendai, Miyagi 980-8579 Japan; 9 Materials Sciences Research Center, Japan Atomic Energy Agency (JAEA), Sayo, Hyogo 679-5148 Japan; 10 Center for Spintronics Research Network, Osaka University, 1-3 Machikaneyama, Toyonaka, Osaka 560-8531 Japan; 11 Forschungszentrum Jülich, PGI-6, 52425 Jülich, Germany

**Keywords:** Physics, Condensed-matter physics, Spintronics

## Abstract

Band-gap engineering is one of the fundamental techniques in semiconductor technology and also applicable in next generation spintronics using the spin degree of freedom. To fully utilize the spintronic materials, it is essential to optimize the spin-dependent electronic structures in the *operando* conditions by applying magnetic and/or electric fields. Here we present an advanced spectroscopic technique to probe the spin-polarized electronic structures by using magnetic circular dichroism (MCD) in resonant inelastic soft X-ray scattering (RIXS) under an external magnetic field. Thanks to the spin-selective dipole-allowed transitions in RIXS-MCD, we have successfully demonstrated the direct evidence of the perfectly spin-polarized electronic structures for the prototypical halfmetallic Heusller alloy $\hbox {Co}_2\hbox {MnSi}$. RIXS-MCD is a promising tool to probe the spin-dependent carriers and band-gap induced in the buried magnetic layers in an element specific way under the *operando* conditions.

## Introduction

Toward the next generation spintronics technology, the manipulation of the spin degree of freedom attracts strong attention. The key parameters to design the functional devices such as the magnetic tunneling junction are the spin-dependent band gap and the spin polarization in the vicinity of the Fermi level ($E_\text {F}$) for the conducting electrons in the magnetic elements. Since the active magnetic layers are usually covered by capping layers in the device, the spin-polarized electronic structures of the buried magnetic systems are important to be clarified by using bulk sensitive spectroscopic techniques.

**Figure 1:**
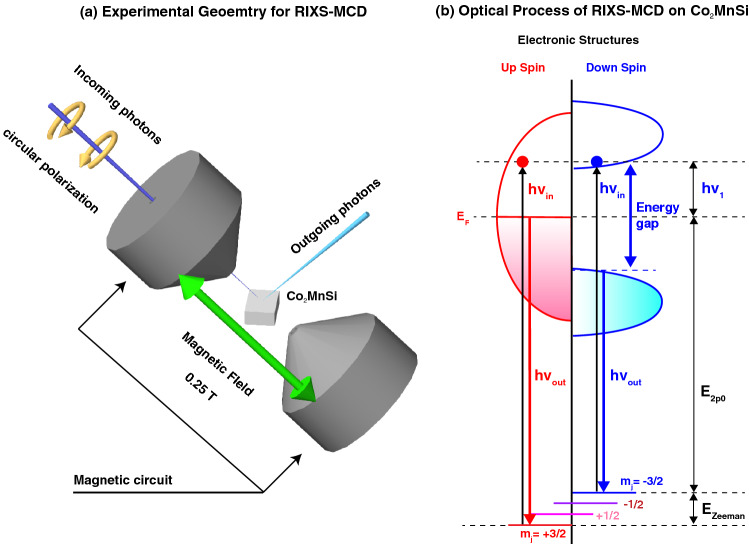
(**a**) Geometry of the RIXS experiments under the magnetic field. The illustration was drawn with Vectorworks 2019 software. (**b**) The optical process of RIXS-MCD. The relative photon energy is defined as $h\nu _{1} = h\nu _{in} - E_{2p_0}$, where $E_{2p_0}$ is the energy difference between the $E_\text {F}$ and $2p_{3/2}$ $m_{j} = -3/2$ states.

Here, we have established a versatile experimental technique to study the electronic structures of the spintronic systems owing to the resonant inelastic X-ray scattering (RIXS) in an external magnetic field. RIXS is a photon-in/out spectroscopy by tuning the incoming photon energy to the core-level absorption edges^1,2^, offering us powerful element-specific probes of the electronic structures even for the buried magnetic layers thanks to the long probing depth (>100 nm)^3^. Moreover, any external perturbations, such as magnetic field, can be applied during the measurements as shown in Fig. 1a^4–6^, giving the great advantage to study the electronic structures under the *operando* conditions for the device application in comparison with other electronic spectroscopies. It has been reported that the magnetic circular dichroism (MCD) in RIXS is effective to probe the magnetic excitations in the magnetically ordered states^5,7^ and the spin-polarized electronic structures of the magnetic materials^6,8^.

**Figure 2:**
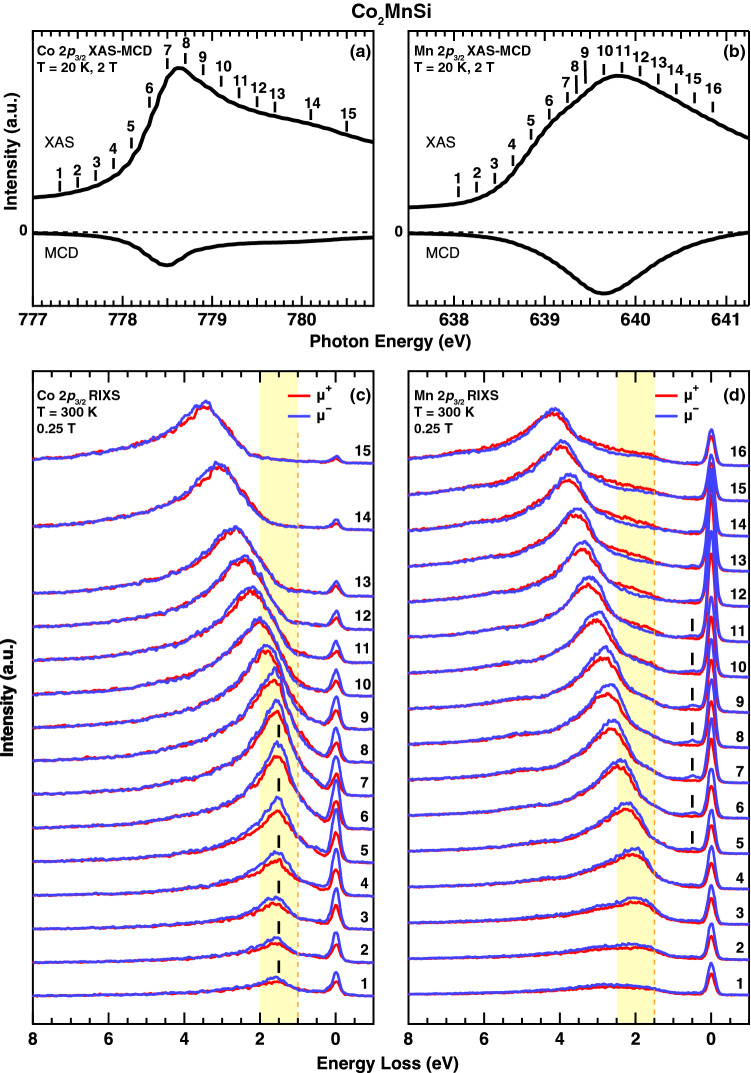
(**a**,**b**) XAS and XAS-MCD spectra of $\hbox {Co}_2\hbox {MnSi}$ obtained at the Co and Mn 2$p_{3/2}$ edges. (**c**,**d**) Photon energy dependence of the Co and Mn 2$p_{3/2}$ RIXS spectra for $\hbox {Co}_2\hbox {MnSi}$ obtained by $\mu ^+$ and $\mu ^-$ configurations recorded at the excitation photon energies indicated by the numbers above the vertical bars on the XAS spectra. The hatched regions are guides to the eye for showing the evolution of the RIXS structures, and the vertical dashed lines indicate the threshold energies.

The RIXS intensity is represented by the Kramers-Heisenberg formula, which is written as

1 $$\begin{aligned} \sigma (\nu _{\mathrm{in}},\nu _{\mathrm{out}})\propto \sum _{b}{\left| \sum _{i}\frac{ \langle b|{\hat{F}}^{(\mu _2)}_2|i\rangle \langle i|{\hat{F}}^{(\mu _1)}_1|a\rangle }{E_{i}-E_{a}-h\nu _{\mathrm{in}}-\mathrm{i}\Gamma _i}\right| }^2 \times \delta [(h\nu _{\mathrm{in}}-h\nu _{\mathrm{out}})-(E_b-E_a)], \end{aligned}$$

where *a*, *b*, and *i* denote the initial, final, and intermediate states having the energy of $E_a$, $E_b$, and $E_i$, respectively, and the lifetime broadening $\Gamma _i$ of the intermediate states. ${\hat{F}}^{(\mu )}$ is the dipole transition operator for the polarization of $\mu _1$ ($\mu _2$) of incoming (outgoing) photon with energy of $h\nu _{in}$($h\nu _{out}$). When the external magnetic field induces the magnetic ordering, the degeneracy of the core levels is lifted by the effective exchange field due to the 3*d* states of the magnetic elements as shown in Fig. 1b. This core-level Zeeman splitting induces the spin-polarized core levels in the 2$p_{3/2}$ with $m{_j} = {\pm }3/2$ states. Then the spin-selective dipole-allowed transitions between the 2$p_{m{_j}=\pm 3/2}$ and 3*d* states by using the circularly polarized photons give the information on the spin-dependent electronic structures in the RIXS-MCD spectra. Note that the transition probabilities for the 2$p_{{\pm }3/2}$ states in both excitation and decay channels are much larger than those for the ${m{_j}={\pm }1/2}$ states^6^, having the spin-degeneracy due to the strong spin-orbit interaction in the 2*p* edges (See “Methods:Theory”).

**Figure 3:**
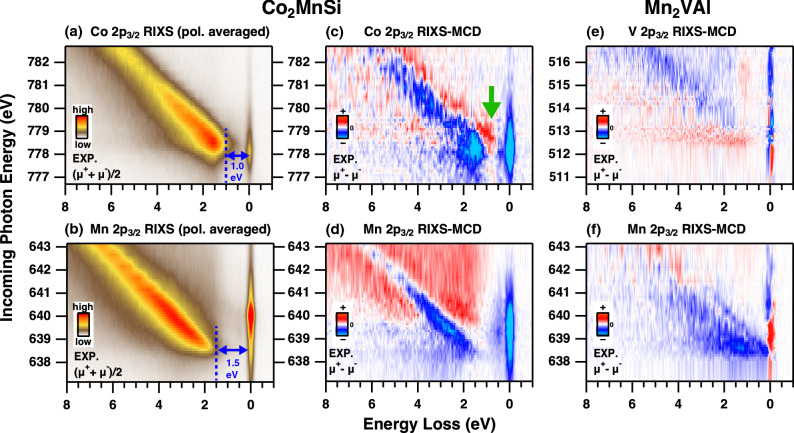
Intensity plot of the RIXS data for $\hbox {Co}_2\hbox {MnSi}$ obtained by the circularly polarized photons. The polarization averaged RIXS at the Co 2$p_{3/2}$ edge (**a**) and at the Mn 2$p_{3/2}$ edge (**b**), and the RIXS-MCD data for the Co and Mn edges in (**c**,**d**), respectively. The RIXS-MCD data for $\hbox {Mn}_2\hbox {VAl}$ obtained at the V and Mn 2$p_{3/2}$ edges are also shown in (**e**,**f**). The gap energies in (**a**,**b**) are estimated by the energy difference between the elastic peak and the threshold of the RIXS structures as shown in Fig. 2c,d.

**Figure 4:**
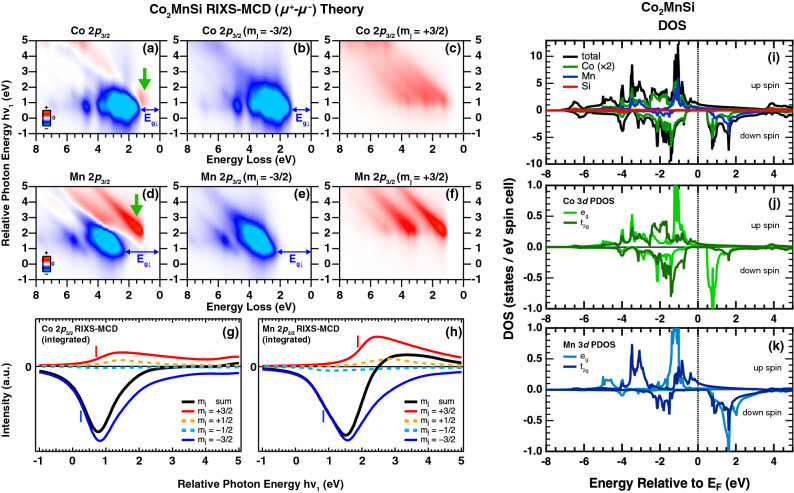
Theoretical RIXS-MCD spectra for $\hbox {Co}_2\hbox {MnSi}$ at the Co 2$p_{3/2}$ edge (**a**). The $m{_{j}} = -3/2$ and $+3/2$ components are shown in (**b**,**c**). Theoretical RIXS-MCD at the Mn 2$p_{3/2}$ edge (**d**) and the $m{_{j}} = \pm 3/2$ components are shown in (**e**,**f**). The green arrows in (**a**,**d**) indicate the $m{_{j}} = +3/2$ components located in the down-spin gap ($E_{g_{\downarrow }}$) of $m{_{j}} = -3/2$ components obtained in (**b**,**e**). The incoming photon energy dependence of the $m{_j}$ resolved MCD intensity integrated over whole loss energies are also shown in (**g**,**h**) for Co and Mn edges, respectively. The vertical bars indicate the threshold energies of the $m{_j} = \pm 3/2$ components. (**i**) the theoretical prediction of the spin-dependent DOS and PDOS of Co, Mn, and Si. (**j**,**k**) the $e_g$ and $t_{2g}$ components of the PDOS of Co and Mn, respectively.

By utilizing this innovative technique, we focus on the prototypical halfmetallic ferromagnet $\hbox {Co}_2\hbox {MnSi}$, which is one of the L2$_1$-type full-Heusler alloys having the up-spin states with 100% spin polarization at $E_\text {F}$ in the down-spin gap as predicted by the density functional theory (DFT)^9^. The Curie temperature of 985 K is high enough for the applications^10,11^, and the giant tunneling magnetoresistance was reported over 300% at 290 K for the epitaxial magnetic tunneling junctions^12,13^. The experimental magnetic moment is close to 5 $\mu _B$/f.u.^10^, which is in agreement with the value expected from the Slater-Pauling rule for the halfmetallic materials^14^. In addition, the anisotropic magnetoresistance (AMR) ratio is negative for the wide range of the relative angle $\theta$ between the magnetization and the electric current directions with negative peaks at $\theta =0^{\circ }$ and $180^{\circ }$, supporting the halfmetallic nature^15,16^. The local electronic structures and magnetic properties have been investigated by using the photoemission spectroscopy and the MCD in X-ray absorption spectroscopy (XAS), and understood as the Co 3*d* electrons have rather itinerant character and the Mn 3*d* electrons contribute noticeably to the local magnetic moment^17–19^. Moreover, the spin-resolved “*unoccupied*” partial density of states (PDOS) were extracted from the XAS-MCD spectra^20,21^.

**Figure 5:**
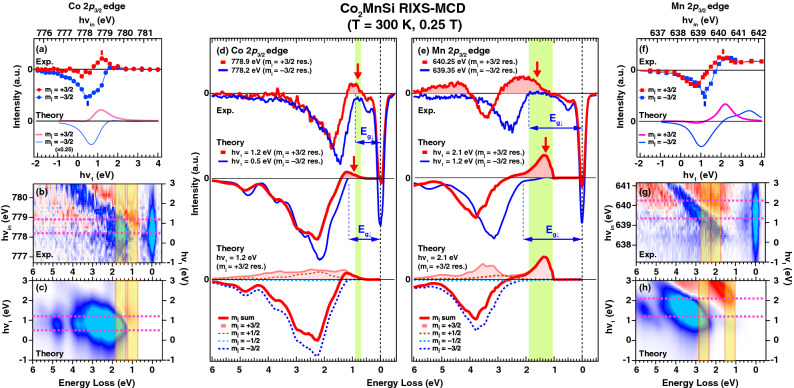
Comparison of the RIXS-MCD spectra between the experiment and theory. Results for the Co(Mn) $2p_{3/2}$ edge are shown in the left (right) half. (**a**,**f**) Incoming photon-energy dependence of the RIXS-MCD intensity of Co and Mn 2$p_{3/2}$ edge at the selected loss energy for $m_{j} = \pm 3/2$ components with full energy window of 0.5 eV as indicated by the yellow-colored hatched area in the intensity plot for experiment (**b**,**g**) and theory (**c**,**h**). The same color scales as in Figs.3 and 4 are employed here. (**d**,**e**) The selected RIXS-MCD spectra for the Co (Mn) edge obtained at the $m_{j} = \pm 3/2$ resonance energies defined by the peak energies as indicated by vertical bars in (**a**,**f**), respectively. The resonance energies for the $m_{j} = \pm 3/2$ components are also shown by the dashed lines in (**b**,**c**,**g**,**h**). To minimize the experimental artifacts, each spectrum was measured for opposite orientation of the external magnetic field and the obtained spectra were averaged. Moreover, the experimental spectra are scaled to discuss the line shape. The experimental data (top curves in (**d**,**e**)) are compared with theory (middle curves) together with the theoretical $m_{j}$ resolved spectra at $m{_{j}} = +3/2$ resonance (bottom curves). The green hatched regions in (**d**,**e**) are the guides to the eye for showing the energy range, where $m_{j} = +3/2$ components are observed in the gap of the $m_{j} = -3/2$ spectra.

Nevertheless, the direct observation of the halfmetallic electronic structures is still challenging. Since the coercivity for the bulk single crystals is small, it was difficult to study the spin polarized electronic structures using the $\textit{standard}$ spin-resolved photoemission (SPES) approach. Thus, only few SPES works have been reported so far for the halfmetallic electronic structures in $\hbox {Co}_2\hbox {MnSi}$ film samples having the remanent magnetization^22^. In addition, it is well known that the magnetic properties and electronic structures of the Heusler alloys are often changed by various disorders^11,23^, and the surface sensitivity of SPES always hinders to probe the electronic structures of the buried magnetic layers after fabricating the device structures. Meanwhile, it has been reported that the RIXS-MCD allows us to probe the spin-polarized electronic structures in ferrimagnetic $\hbox {Mn}_2\hbox {VAl}$^6^, but the halfmetallic gap was not directly observed in the Mn $2p_{3/2}$ edge since the spin-gap opens on the up-spin side in $\hbox {Mn}_2\hbox {VAl}$. In order to experimentally prove the halfmetallic nature, it is important to detect the spin-polarized electronic structures across the Fermi level observed in the gap of opposite spin. This motivated us to perform the RIXS-MCD experiments for the high-quality single crystal of ferromagnetic $\hbox {Co}_2\hbox {MnSi}$, which has the expected halfmetallic gap on the down-spin side, under the magnetic field.

## Results

Figure 2a,b show the Co and Mn $2p_{3/2}$ XAS and its MCD spectra obtained by the total electron yield mode recorded at 20 K, where the numbers indicated above the XAS spectra show the excitation photon energies of the RIXS spectra in Fig. 2c,d, respectively. The RIXS spectra measured in parallel and antiparallel configurations between the light helicity and the direction of the magnetic field, denoted as $\mu ^+$ and $\mu ^-$, show the circular polarization dependence for both the Co and Mn $2p_{3/2}$ edges. In the Co edge, the RIXS peak structure develops at the constant energy loss around 1.5 eV (indicated by solid vertical bars in Fig. 2c) with increasing the excitation photon energies from the pre-edge to the vicinity of the peak top of the XAS (labeled from 1 to 8), suggesting the localized character of the *dd*-excitations on the Co site. Above this photon energy of 8 (778.7 eV), the energy loss feature shift linearly with the excitation energies, suggesting the fluorescence feature due to the decay from the shallow occupied states to the empty core hole states, which reflects the itinerant character of the unoccupied states in the intermediate states. On the other hand, the Mn $2p_{3/2}$ RIXS spectra show the fluorescence peak around 2.0 eV in the pre-edge region, which shifts linearly with increasing the photon energy. This indicates that the character of the Mn 3*d* states is much different from that of the Co 3*d* states. One should, however, notice the small energy loss feature at around 0.5 eV for the excitation photon energies between 5 and 11 for the Mn $2p_{3/2}$ RIXS as indicated by solid vertical bars in Fig. 2d.

The detailed intensity map of the polarization averaged RIXS spectra obtained by ($\mu ^+$+$\mu ^-$)/2 for the Co and Mn $2p_{3/2}$ edges are displayed in Fig. 3a,b, respectively. The fluorescence components reflecting the occupied electronic structures of the 3*d* states are dominant for both Co and Mn edges with the energy gap from the elastic line. This gap size for the Co edge is 1.0 eV, which is smaller than that for the Mn edge of 1.5 eV. This reflects the displacement of the upper edge of the occupied PDOS of the valence band electronic structures between Co and Mn 3*d* states.

The RIXS-MCD spectra given by $\mu {^+}-\mu {^-}$ are displayed in Fig. 3c,d for the Co and Mn $2p_{3/2}$ edges, respectively. The MCD contrast is clearly observed especially for the fluorescence components, and the negative MCD signals show up on the lower excitation energy side than the positive MCD signals. The negative MCD in the Co edge first shows rather constant energy-loss features at 1.5 eV around $h\nu _{in}$ of 778 eV, suggesting the local *dd*-excitation with the energy gap of $\sim 1.0$ eV from the elastic peak. An additional low energy excitation with constant energy-loss at 0.5 eV shows negative MCD signals for both Co and Mn edges, suggesting the possible magnetic excitation as discussed later^24,25^. Most strikingly, the positive MCD signals on the Co edge (indicated by the downward green arrow in Fig. 3c) show up around 1 eV of loss energy *in the gap* of the negative MCD signals, which is also confirmed by the RIXS spectra in Fig. 2c. On the other hand, the RIXS-MCD on the Mn edge in Fig. 3d shows the negative fluorescence MCD with the gap of 1.5 eV from the elastic line. This feature is clearly different from the Mn RIXS-MCD for halfmetallic ferrimagnet $\hbox {Mn}_2\hbox {VAl}$, which shows that the negative fluorescence-MCD signals branch off from the elastic line at the beginning of the Mn $2p_{3/2}$ resonance in Fig. 3f (as discussed later)^6,9,26–28^.

In order to discuss the electronic structures responsible for the RIXS-MCD, we have simulated the RIXS-MCD spectra based on the Kramers-Heisenberg formula with practical assumption that the square of the matrix element in the numerator is proportional to the PDOS of the involved occupied and unoccupied states multiplied by the transition probabilities as formulated in Eq. 2, (see “Methods:Theory”). Note that the XAS and the valence band hard-X-ray photoemission (HAXPES) spectra are qualitatively described by the DOS-based simulations, (see Supplementary materials), so that the DOS obtained by DFT reasonably explains the electronic structures of $\hbox {Co}_2\hbox {MnSi}$. The simulation of the RIXS-MCD spectra for the Co edge in Fig. 4a shows the dominant negative MCD starting around the loss energy of 1.5 eV and noticeable positive MCD observed around 1.0 eV. The excitation energy is expressed by the relative photon energy defined as $h\nu _{1}= h\nu _{in} - E_{2p_0}$, which is the difference of the photon energy $h\nu _{in}$ from the energy $E_{2p0}$ between the $E_\text {F}$ and the $m{_j}=-3/2$ core state as shown in Fig. 1b. We should point out that $h\nu _1$ for the positive MCD in Fig. 4a is about 1 eV higher than that for the negative MCD, which is consistent with the experimental results in Fig. 3c. The MCD contrast is mainly due to the optical transition between the 2*p* core levels with magnetic quantum number $m{_j} = \pm 3/2$ lifted by the Zeeman splitting due to the effective magnetic field from the 3*d* states (see Supplementary materials). Since the $2p_{\pm 3/2}$ states are fully spin polarized, the spin-selective transitions take place by the spin-polarized electrons. Namely, negative (positive) RIXS-MCD signals reflect the Co 3*d* states with spin-down (-up) components. This was explicable by the theoretical simulations resolving the $m{_j}$ components as shown in Fig. 4b,c. The negative MCD mainly originates from $m{_j} = -3/2$ contributions with the gap size of $\sim 1.2$ eV (Fig. 4b), which is consistent with the down-spin band gap of the PDOS of Co 3*d* states as shown in Fig. 4i,j. Most interesting is that $m{_j} = +3/2$ contributions with up-spin character are observed in the down-spin gap ($E_{g_{\downarrow }}$) as indicated by the green downward arrow in Fig. 4a, in consistence with the halfmetallic electronic structures of the Co 3*d* states as illustrated in Fig. 1b.

The simulated spectra for the Mn edge are also qualitatively consistent with the experimental results, and again the down-spin gap is also observed for the $m{_j} = -3/2$ contributions as shown in Fig. 4d,e. The gap size of the down-spin contribution is about 2.2 eV in Fig. 4e, which is wider than that of the Co 3*d* states in agreement with the PDOS as shown in Fig. 4k. Moreover, peaks at the Mn edge shift linearly with the incoming photon energy in Fig. 4d,e, whereas peaks at the Co edge in Fig. 4a,b show rather constant energy-loss feature, reflecting the localized character of the Co 3*d* states from the beginning of the resonance to the $h\nu _1$ of about 1.0 eV. According to Eq. 2, the difference of the $h\nu _1$-dependence of the Co and Mn edges comes from the electronic structures of the unoccupied 3*d* states. As shown in Fig. 4j,k, the band-width of the unoccupied Mn 3*d* states is wider than that of the Co 3*d* states with the relatively sharp unoccupied PDOS near the bottom of the conduction band in the down-spin case, which is mainly dominated by the anti-bonding $e_g$ states originating from the Co 3*d* - Co 3*d* hybridization between the neighboring Co sites^14^. Therefore, the incoming photon energy dependence of the RIXS-MCD at the Co edge reflects localized character of unoccupied states of the Co 3*d* states, while that at the Mn edge shows rather itinerant character of the unoccupied Mn 3*d* states. On the other hand, occupied PDOSs show prominent difference between the Co and Mn. Namely, $e_g$ states as well as up-spin $t_{2g}$ states of Mn are much more localized than those of Co as recognized in Fig. 4j,k. Therefore, the RIXS-MCD spectra show the strong contrast between the positive and negative signals due to the $m{_j} = +3/2$ and $-3/2$ components as shown in Fig. 4d–f.

The core-level Zeeman splitting, which is one of the most important parameters for the spin-selective excitation, can be estimated by the energy difference between the $m{_j} = \pm 3/2$ components. To examine the estimation of the splitting energy, the $m{_j}$ resolved simulations for all $m{_j}$ components of $2p_{3/2}$ states are integrated along the loss energy axis as shown in Fig. 4g,h for Co and Mn edges. The difference of the two threshold energies of the $m{_j} = \pm 3/2$ components, estimated at the half maximum of their MCD peaks as indicated by vertical bars, yields 0.5 and 1.0 eV for Co and Mn edges, representing the Zeeman splitting (see “Methods:Theory”). The good agreement with the experimental results indicates that the calculated values of Zeeman splitting were reasonable, and also confirms the validity of the simulation method. Note that the $m{_j} = \pm 1/2$ contributions, which cause spin-flip (non spin-conserving) excitations due to the spin degeneracy from the strong spin-orbit coupling (SOC) of the 2*p* states, contribute much less to the RIXS-MCD than those of the $m{_j} = \pm 3/2$ components, (see Supplementary materials). This is due to the difference of the transition probabilities of the dipole-allowed transitions, which are applied for both excitation and decay channels in the RIXS process.

Figure 5a upper panel shows the incoming photon-energy dependence of the experimental RIXS-MCD intensity of the Co 2$p_{3/2}$ edge integrated within 0.5 eV full width ($\pm 0.25$ eV) around the energy loss of 1.6 eV (1.0 eV) corresponding to the $m_{j} = -3/2$ (+3/2) component as shown by yellow hatched regions in Fig. 5b. The peak of resonance for the $m_{j} = -3/2$ contribution is located at 778.2 eV ($h\nu _{1}=0.5$ eV), and the delayed resonance of $m_{j} = +3/2$ components is observed at 778.9 eV ($h\nu _{1}=1.2$ eV) due to the core-level Zeeman splitting. The resonance energies for both $m_{j} = \pm 3/2$ components are consistent with the theoretical simulations as shown in the lower panel of Fig. 5a, which are extracted from the results in Fig. 5c. The experimental RIXS-MCD spectra obtained at the photon energies corresponding to the $m_{j} = \pm 3/2$ resonance peaks in Fig. 5a are displayed in the top panel of Fig. 5d. The $m_{j} = -3/2$ spectrum recorded at 778.2 eV shows the down-spin gap $E_{g_{\downarrow }}$ of 0.9 eV estimated from the onset of the fluorescence MCD signals (see Supplementary materials), which is comparable to the simulation in the middle panel as well as PDOS of the Co 3*d* states as shown in Fig. 4j. On the other hand, the RIXS-MCD spectrum obtained at the $m_{j} = +3/2$ resonance at 778.9 eV (top panel in Fig. 5d) shows the positive MCD signals around 1 eV, which is consistent with the theoretical simulation at $h\nu _{1}$ of 1.2 eV (middle panel in Fig. 5d). These positive MCD signals are mainly due to the $m_{j} = +3/2$ contributions with the up-spin components as shown in the $m_{j}$-resolved spectra obtained at $h\nu _{1}$ of 1.2 eV in the bottom panel of Fig. 5d. Most important is that the up-spin components are observed in the down-spin gap as shown in Fig. 1b. Since the Co 3*d* states contribute mainly to the electronic structures in the vicinity of the $E_\text {F}$ as supported by the valence band HAXPES (see Supplementary materials), the up-spin components in the down-spin gap contribute to the electronic states at the $E_\text {F}$, proving the halfmetallic electronic structures of the Co 3*d* states. Note that the low energy excitation around 0.5 eV (top panel of Fig. 5d) is not observed in the DOS-based theory, and thus it is possibly due to the spin wave excitations predicted by the theoretical simulations^24,25^.

In the same manner, the behavior of the spin-polarized electronic structures of the Mn 3*d* states are shown in the top panel of Fig. 5e obtained at the $m_{j} = \pm 3/2$ resonance energies (640.25 and 639.35 eV) determined from the photon energy dependence of the RIXS intensity in Fig. 5f, which is extracted from Fig. 5g,h. The down-spin gap $E_{g_{\downarrow }}$ of 1.9 eV is comparable to the energy gap of 1.7 eV in the Mn 3*d* PDOS as shown in Fig. 4k, and the positive MCD signals reflecting the up-spin contributions of the Mn 3*d* states in the vicinity of the $E_\text {F}$ are also observed in the gap as in the case of Co. This provides the direct evidence of the presence of the up-spin states in the down-spin gap, i.e. the halfmetallic electronic structures for $\hbox {Co}_2\hbox {MnSi}$, in line with the AMR measurements^15,16^ and the previous spectroscopic investigations using SPES^22^ and XAS-MCD^20,21^ for the *occupied* and *unoccupied* electronic structures, respectively. RIXS-MCD is a powerful technique to probe the spin-polarized electronic structures for both *occupied* and *unoccupied* side with element specific way, giving the great advantage to investigate the spin-dependent gap in the halfmetallic materials.

## Discussion

We should point out the difference of the RIXS-MCD spectra for the Mn edge between ferromagnetic $\hbox {Co}_2\hbox {MnSi}$ and ferrimagnetic $\hbox {Mn}_2\hbox {VAl}$ as shown in Fig. 3d,f, respectively. Since the halfmetallic gap of $\hbox {Mn}_2\hbox {VAl}$ opens in the up-spin side (see Supplementary materials)^9,28^, the negative RIXS-MCD signals of $\hbox {Mn}_2\hbox {VAl}$ can branch off from the elastic line due to the spin polarized electronic structures for the spin-down subband around the $E_\text {F}$^6^. Moreover, the antiparallel spin coupling between Mn and V in ferrimagnetic $\hbox {Mn}_2\hbox {VAl}$^28^ induces the opposite order of the core-level Zeeman splitting for Mn and V 2$p_{3/2}$ states (see Supplementary materials). This attributes to the reversed RIXS-MCD contrast between Mn and V edges of $\hbox {Mn}_2\hbox {VAl}$^6^ as shown in Fig. 3f,e, and the gap of the $m_{j} = +3/2$ components in the V 2$p_{3/2}$ edge around 512.5 eV in Fig. 3e reflects the up-spin band gap of the V 3*d* PDOS of $\hbox {Mn}_2\hbox {VAl}$ (see Supplementary materials). This is practically important that RIXS-MCD directly probes which spin subbands opens the halfmetallic gap as observed in the difference between $\hbox {Co}_2\hbox {MnSi}$ and $\hbox {Mn}_2\hbox {VAl}$, giving the important information to design the spintronic devices. Since the transition probability of the spin-polarized $m_{j} = \pm 3/2$ states are generally larger than that for the $m_{j} = \pm 1/2$ states, this technique can be adopted for the broad range of the spintronic materials.

To summarize, we have successfully established the method to probe the spin-polarized electronic structures using RIXS-MCD in the magnetic field. The potential of RIXS-MCD is not only for detecting the spin-polarized electronic structures, but also for confirming the spin-dependent gap even for the capped samples under the *operando* conditions. Moreover, the element specific characteristics are so powerful for clarifying the electronic structures of the buried device structures such as magnetic tunneling junction constituted by several magnetic elements. It will be possible to semi-quantitatively analyze the density of the spin-polarized carriers induced in the heterojunctions under the *operando* conditions by applying the magnetic and/or electric fields. Therefore, we stress that RIXS-MCD is a very suitable technique for acquiring the necessary information for designing the spintronics devices, opening up a new field of science.

## Methods

### Single crystalline growth and sample characterization

Mother ingot of polycrystalline $\hbox {Co}_2\hbox {MnSi}$ was fabricated by arc melting in an argon gas atmosphere. Since the vapor pressure of Mn is high during the melting, excess Mn elements are contained in the mother ingot. Single crystal was grown by the Bridgeman method with a size of 12 mm in diameter and about 30 mm in length. The obtained ingot was annealed at 1373 K to enlarge the crystal grains and then slowly cooled down to the room temperature. Crystal orientation was checked by the back Laue method and the specimen was cut in a strip form in the direction being parallel to $\langle 100\rangle$. The sample composition was evaluated to be Co: 50.0, Mn: 25.9, Si: 24.1 (atomic %) with an electron probe microanalyzer. The electronic structures are, however, robust for the slight off-stoichiometric effect in the present case, which is simulated by the density functional theory, (see Supplementary materials). Sample magnetization was measured with a superconducting quantum interference devices (SQUID) magnetometer. Saturation magnetic moment evaluated from the magnetization curve at 5 K for $\hbox {Co}_2\hbox {MnSi}$ was 142.2 emu/g and converted to 5.1 $\mu _B$/f.u. The expected value of the magnetic moment for $\hbox {Co}_2\hbox {MnSi}$ by Slater-Pauling rule is 5 $\mu _B$/f.u.^14^, being consistent with the experimental value. The Curie temperature of the present specimen detected by differential scanning calorimetry is 1020 K, being also comparable to the literature^10,11^.

### RIXS experiment

RIXS measurements were performed at room temperature at SPring-8 BL07LSU HORNET end-station, where either linearly or circularly polarized light could be delivered^29,30^. For RIXS-MCD measurements using left and right circularly polarized light, the external magnetic field of 0.25 T was applied by a magnetic circuit with a permanent magnet, which has two poles with holes for passing the excitation light through^4,5^. The magnetic circuit was mounted on the rotational feed through, and the direction of the magnetic field was reversed to make sure the genuine magnetic signals^4^. The total energy resolution was set to $\sim 200$ meV.

### Theory

The electronic structure calculations based on DFT are performed using the HiLAPW code, the all-electron full-potential linearized augmented plane-wave (FLAPW) method^31^. The generalized gradient approximation (GGA) using the Perdew-Burke-Ernzerhof scheme^32^ is used for the exchange-correlation potential. Here the full relativistic effects including the SOC are taken into account for the Co and Mn 2*p* core states. On the other hand, the spin-orbit coupling for the 3*d* states is negligible compared with the 2*p* states, since the orbital magnetic moments per hole of Co and Mn were estimated as 0.009$\mu _B$/Co/hole and 0.002$\mu _B$/Mn/hole by means of the sum-rule analysis for the XAS-MCD spectra, respectively, (see Supplementary materials). Plane-wave expansion cutoffs are set to 20 Ry for the wave functions and 160 Ry for the charge density and potential functions. For the Brillouin-zone integration, a $16\times 16\times 16$ uniform mesh is used with the tetrahedron integration technique. The obtained DOS well explain the valence band photoemission and XAS spectra, (see Supplementary materials).

The calculated total magnetic moment is 5.00 $\mu _{\mathrm{B}}$, which is a perfectly integer value resulting from the halfmetallicity. The calculated spin magnetic moments of the Co and Mn 3*d* states are 1.05 $\mu _{\mathrm{B}}$ and 2.80 $\mu _{\mathrm{B}}$, respectively. These exchange spilt 3*d* states under external magnetic field cause the Zeeman splitting of the 2*p* core states. The magnitudes of their Zeeman splitting of $2p~j=3/2$ states are about 0.5 eV for the Co atom and 1.0 eV for the Mn atom, and their states are energetically higher in the order of $m_{j}=-3/2,-1/2,+1/2,$ and $+3/2$. Figure 4j,k show the calculated PDOS of the Co and Mn 3*d* orbitals.

The RIXS spectra are simulated based on the Kramers-Heisenberg formula written as eq. 1^1,2,6^. If it is assumed that the square of the matrix element in the numerator is proportional to the PDOS of the involved occupied (unoccupied) states described as $D^{\mathrm{occ}}$ ($D^{\mathrm{unocc}}$) at the energy of $\varepsilon _{v}$ ($\varepsilon _{e}$) of occupied (unoccupied) valence states, this formula can be translated to

2 $$\begin{aligned} \sigma ^{\mu _1,\mu _2}(\nu _1,\nu _{\mathrm{out}})\propto \sum _{jm_j}\sum _{m_s,m'_s}\int d\varepsilon _{v^{-1}}\frac{w^{(\mu _2)}_{jm_jm'_s}w^{(\mu _1)}_{jm_jm_s}D^{\mathrm{occ}}_{(m'+\mu _2)m'_s}(\varepsilon _{v^{-1}})D^{\mathrm{unocc}}_{(m+\mu _1)m_s}(h\nu _1+E_{\mathrm{F}})}{(\varepsilon _{v^{-1}}-E_{2p_{jm_j}}-h\nu _{\mathrm{out}})^2+\Gamma _i^2} \end{aligned}$$

by the density of states (DOS) approximation, considering electric dipole transition probabilities of the incoming (outgoing) photon helicity $\mu _{1}(\mu _{2})$. The weight coefficient $w^{(\mu )}_{jm_jm_s}$ is a square of product of Clebsch-Gordan coeffcient and the Gaunt coefficient. Here, *j*, $m_j$, and $m_s$ are the total angular momentum of the 2*p* core states, their *z* component, and the spin of the 3*d* states, respectively. For the 2*p* states, *m* is given by $m_{j}-m_{s}$. The energy of unoccupied valence states $\varepsilon _{e}$ using the offset energy ($h\nu _{1}$) relative to the $E_{\mathrm{F}}$ is written as $\varepsilon _{e} = E_{\mathrm{F}} + h\nu _1$, and $\varepsilon _{v^{-1}}$ stand for the energy of valence electron with one hole. $h\nu _{\mathrm{out}}$ denotes the outgoing photon energy. The lifetime broadening $\Gamma _i$ values are set to 0.47 (0.36) eV for the Co (Mn) $L_3$-edges^33^. Although all one electron excitations are considered in equation 2, such excitations as the *dd*-excitations originating from the multiplet effects as well as magnon excitations are not handled there.

Note that the transition probabilities depending upon the helicity of photon are considered only in the absorption process, but they are averaged in the emission process since the polarization of the outgoing photon is not observed in the measurement. The SOC of the 3*d* states is neglected because it is much smaller than that of the 2*p* core states and is not thought to affect the RIXS spectra noticeably. Due to spin degeneracy from the strong SOC, the $m_j=+1/2$ and $-1/2$ states of the 2*p* core electrons cause spin-flip (non spin-conserving) excitations.

## Supplementary information

Supplementary Information.
