# Supplementary material for: Detecting halfmetallic electronic structures of spintronic materials in a magnetic field
Source: Sci Rep. 2021 Sep 20;11:18654. doi: 10.1038/s41598-021-97992-z (PMC8452713; doi:10.1038/s41598-021-97992-z)
Supplement: Supplementary file 1 — Supplementary Information. [file 41598_2021_97992_MOESM1_ESM.pdf]

Supplementary materials : Detecting halfmetallic electronic structures of spintronic materials in a magnetic field

H. Fujiwara,[1,*] R.Y. Umetsu,[2,3,4] F. Kuroda,[5] J. Miyawaki,[6] T. Kashiuchi,[1] K. Nishimoto,[1] K. Nagai,[1] A. Sekiyama,[1] A. Irizawa,[5] Y. Takeda,[7] Y. Saitoh,[7] T. Oguchi,[5,8] Y. Harada,[6] and S. Suga[5,9]

[1]*Division of Materials Physics, Graduate School of Engineering Science, Osaka University, Toyonaka, Osaka 560-8531, Japan*

[2]*Institute for Materials Research, Tohoku University, 2-1-1 Katahira, Sendai 980-8577, Japan*

[3]*Center for Spintronics Research Network, Tohoku University, 2-1-1 Katahira, Sendai 980-8577, Japan*

[4]*Center for Science and Innovation in Spintronics, 2-1-1 Katahira, Sendai 980-8577, Japan*

[5]*Institute of Scientific and Industrial Research, Osaka University, 8-1 Mihogaoka, Ibaraki 567-0047, Japan*

[6]*Institute for Solid State Physics, The University of Tokyo, 5-1-5 Kashiwanoha, Kashiwa, Chiba 277-8581, Japan, and Synchrotron Radiation Research Organization, The University of Tokyo, 1-1-1 Koto, Sayo-cho, Sayo, Hyogo 679-5148, Japan*

[7]*Materials Sciences Research Center, Japan Atomic Energy Agency (JAEA), Sayo, Hyogo 679-5148, Japan*

[8]*Center for Spintronics Research Network, Osaka University, 1-3 Machikaneyama, Toyonaka, Osaka 560-8531, Japan*

[9]*Forschungszentrum Jülich, PGI-6, 52425 Jülich, Germany*

(Dated: June 22, 2021)

XAS-MCD AND SUM-RULE ANALYSIS

In order to characterize the element specific electronic structures and the magnetic properties, we have performed XAS and XMCD measurements on Co_2MnSi at BL23SU in SPring-8 [1]. The spectra were recorded in total-electron-yield mode with an energy resolution better than 0.1 eV using a superconducting magnet in fields up to 2 T along the incident beam direction. To eliminate any experimental artifacts arising from system errors, each XMCD spectrum was measured for opposite applied magnetic field and the resulting spectra were averaged. The measurement temperature was set to 20 K. Single crystalline samples were fractured *in situ* in ultrahigh vacuum to obtain the clean surface.

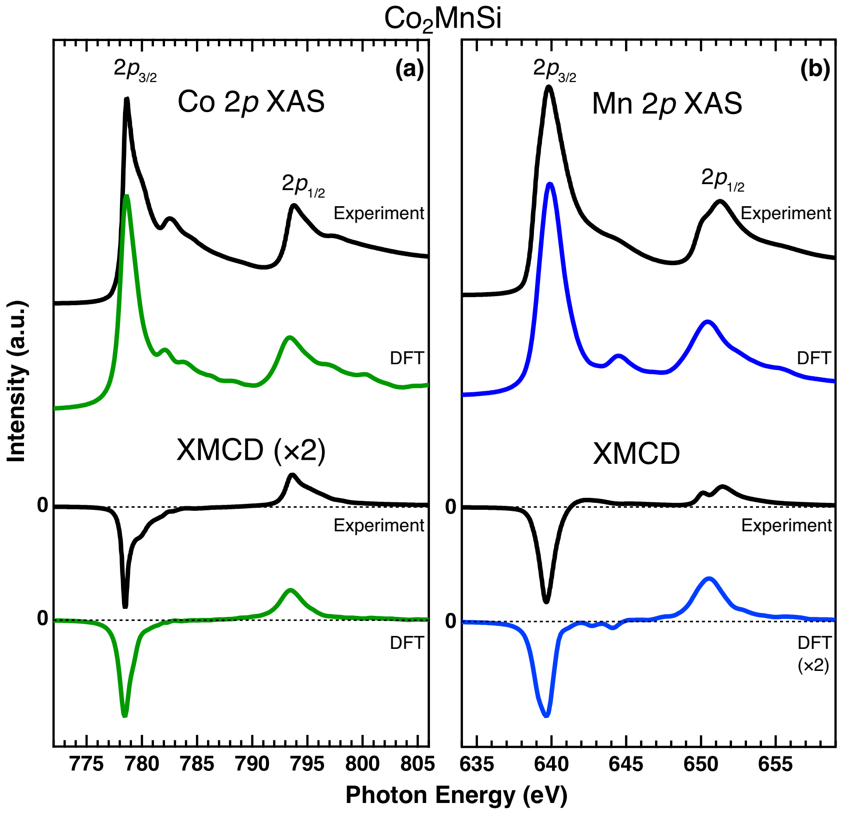

FIG. S1. The XAS and XMCD spectra on Co_2MnSi compared with theoretical simulations based on the DFT for Co and Mn $2p$ edgs in (a) and (b), respectively.

An overview of the Co and Mn $2p$ XAS and XMCD spectra of Co_2MnSi are displayed in Fig, S1(a) and (b), respectively. The spectral line shapes for both Co and Mn edges are well described by the theoretical simulations based on the DFT (GGA). Note that the

double-peak structure in the Mn $2p_{1/2}$ edge of the experimental XMCD spectrum is due to a multiplet structure, indicating a degree of localized character of the Mn $3d$ states [2]. To reveal more detailed magnetic properties of the transition metal sites, the orbital and spin magnetic moments per d hole were estimated by applying the magneto-optical sum-rule [3–5] using the equations,

$$\frac{m_{spin}}{n_h} = -\frac{6p-4q}{rP_c}C, \tag{1}$$

$$\frac{m_{orb}}{n_h} = -\frac{4q}{3rP_c}, \tag{2}$$

where n_h is the number of unoccupied d holes, p (q) is the integral of the XMCD signal over the $L_3(L_{2,3})$-edge, and r is the integral of the polarization summed XAS intensity after subtracting the background. In equations (1) and (2) P_c denotes the degree of circular polarization of 0.97 [1]. C in the spin sum rule is the so-called correction factor to take into account the mixing effect between the $2p_{3/2}$ and $2p_{1/2}$ peaks [6].

Using the orbital sum-rule, we obtained an orbital magnetic moment per hole for Co of $m^{Co}_{orb}/n_h = 0.009$ μ_B/Co/hole, and that for Mn of $m^{Mn}_{orb}/n_h = 0.002$ μ_B/Mn/hole, which are consistent with the estimated values from the DFT, as given in Table I. Assuming $n_h = 3.30$ (5.22) for Co (Mn) estimated from the DFT, we obtained m^{Co}_{orb} (m^{Mn}_{orb}) of 0.03 μ_B/Co (0.01 μ_B/Mn). The spin-orbit coupling of the $3d$ states for Co and Mn is found to be negligibly small, and thus it is not included in the simulation of the RIXS spectra. This allows us to interpret the RIXS-MCD as an effective probe of the spin polarized electronic structures of the $3d$ states using the spin-selective transition. On the other hand, the spin magnetic moment for Co and Mn is derived by assuming $C = 1$. Then, we obtain a spin magnetic moment per hole for Co as $m^{Co}_{spin}/n_h = 0.38$ μ_B/Co/hole, and that for Mn as $m^{Mn}_{spin}/n_h = 0.47$ μ_B/Mn/hole, which are also comparable to the estimated value from DFT, as given in Table I. By multiplying n_h, m^{Co}_{spin} (m^{Mn}_{spin}) is estimated as 1.27 μ_B/Co (2.45 μ_B/Mn), yielding a total moment of 4.99 μ_B. If we use C = 1.5 for Mn [7–9], the total moment is estimated as 6.21 μ_B, giving an error bar of the sum-rule analysis of $\sim$ 25%. The estimated values of the total moment obtained for both C = 1 and 1.5 are comparable for the value of 5.09 μ_B/f.u. obtained by SQUID within the error bar.

Co_2MnSi				
	m_{spin}/n_h (XMCD)	m_{spin}/n_h (DFT)	m_{orb}/n_h (XMCD)	m_{orb}/n_h (DFT)
Co	0.38	0.32	0.009	0.007
Mn	0.47	0.56	0.002	0.002

TABLE I. Spin and orbital magnetic moments of Co_2MnSi in unit of μ_B per hole for an Co (Mn) site obtained by DFT and the sum-rule analysis for experimental XMCD spectra assuming the correction factor $C = 1$.

VALENCE BAND PHOTOEMISSION : LINEARLY-POLARIZED HARD X-RAY PHOTOEMISSION

In order to analyze the RIXS-MCD spectra by the DFT-based simulation, it is important to check the validity of the DOS using the valence-band photoemission spectra. We have performed the hard x-ray photoemission (HAXPES) with $\sim$ 8 keV photons in order to discuss the detailed valence band electronic structures on Co_2MnSi. The HAXPES measurements were carried out at BL19LXU of SPring-8, where the MBS A1-HE hemispherical electron-energy analyzer was installed. The total energy resolution was set to 400 meV at the measurement temperature of 5 K. The photon energy was set to $\sim$7900 eV, and the linear polarization of the X-ray was transformed from the horizontal (p-polarization) to the vertical polarization (s-polarization) by means of the transmission-type diamond phase retarder as shown in Fig. S2(a) [10]. The degree of linear polarization of the polarization switched X-ray was estimated as -0.96, corresponding to the vertically linear polarization component of 98%.

Figure S2(b) shows the valence band HAXPES spectrum obtained by using the linearly polarized photon with s-polarization configuration in order to focus on the $3d$ components in the valence band [10–12]. The HAXPES spectra are qualitatively described by the simulation based on the weighted sum of the partial DOS (PDOS) by taking into account the asymmetry factors of the photoionization cross-section as shown in the bottom of Fig. S2(b) [13–15], especially for the -1.2 eV peak is mainly due to the Co and Mn $3d$ states as indicated by the dashed line. Moreover, both Co and Mn $3d$ states mainly contribute to the electronic

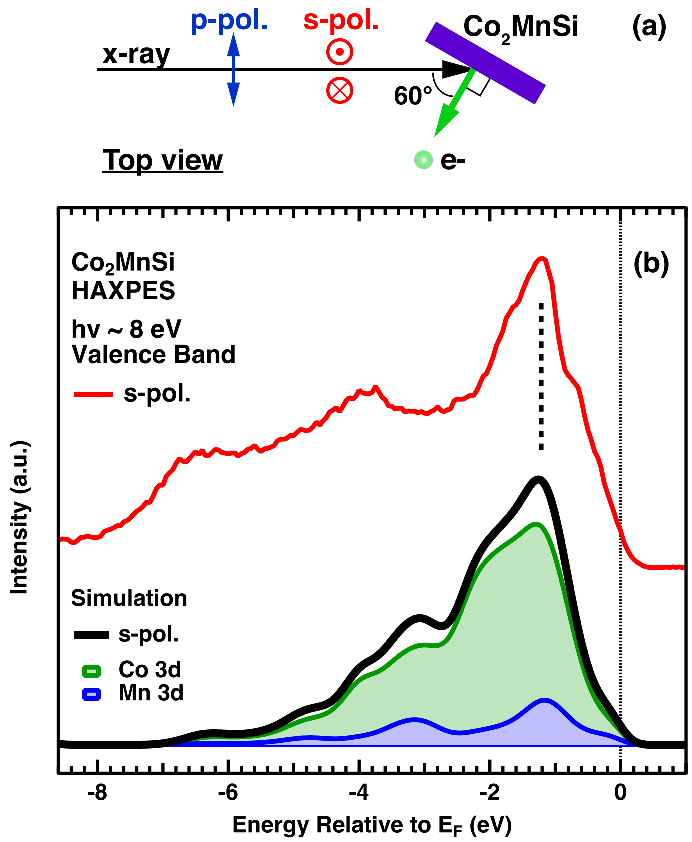

FIG. S2. (a) Experimental geometry of the HAXPES measurements. (b) Valence-band HAXPES spectrum of Co_2MnSi obtained by the s-polarization configuration (top) and the results of theoretical simulation obtained by the DFT (bottom).

structures in the vicinity of the the Fermi level (E_F). These results indicate that the DFT well captures the electronic structures of Co_2MnSi, and support the simulation of the RIXS-MCD spectra based on the DFT.

CALIBRATION OF THE INCOMING PHOTON ENERGY BETWEEN THE EXPERIMENT AND THEORY

In order to calibrate the excitation photon energies for RIXS experiments, the photon energies of the XAS-MCD spectra recorded before the RIXS experiments at BL07LSU in SPring-8 was calibrated by using the negative peak of the XAS-MCD spectra recorded at the BL23SU in SPring-8 as shown in Figs. S3 (a) and (b) for Co and Mn $2p_{3/2}$ edges, respectively. The relative excitation energies of the theoretical simulations were also calibrated by the

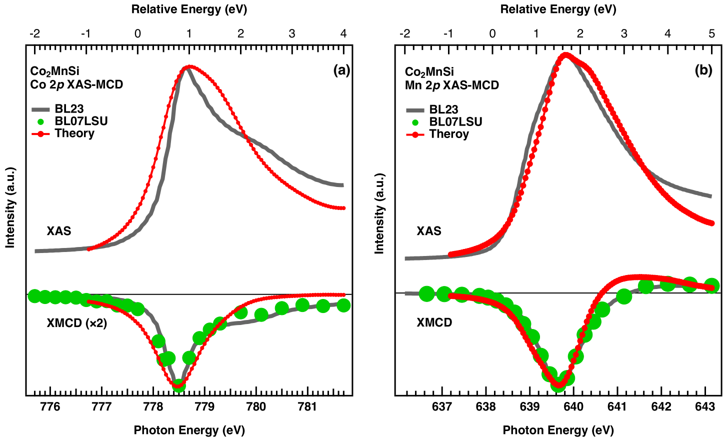

FIG. S3. Comparison of the XAS and XAS-MCD experimental spectra with the theoretical prediction for Co (a) and Mn (b) $2p_{3/2}$ edges. The XAS (upper panel) and MCD (lower panel) spectra are normalized by the peak top.

same manner as the RIXS experiments by using the integrated intensity along the energy loss axis.

THEORETICAL SIMULATIONS OF THE RIXS-MCD

The complete set of the intensity plot of the theoretically simulated RIXS-MCD spectra for Co_2MnSi and Mn_2VAl are displayed in Figs. S4 and S5, respectively. The total RIXS-MCD intensities are resolved into all components of the $m_j = -3/2, -1/2, +1/2, +3/2$ states with the same color scale. The RIXS intensity is dominated by the $m_j = \pm 3/2$ components with the spin-selective transitions. On the other hand, the $m_j = \pm 1/2$ components are composed of both spin-up and -down states due to the spin-orbit coupling. Then the hole spin can be relaxed before the fluorescence decays into the core-hole states with $m_j = \pm 1/2$. However, the intensity of the $m_j = \pm 1/2$ components in the RIXS-MCD is much smaller than that of the $m_j = \pm 3/2$ components, since the transition probabilities for the $m_j = \pm 3/2$ states are much bigger than that for the $m_j = \pm 1/2$ states in the dipole-allowed transition [5]. This is supported by the comparison of the incoming photon energy dependence of the the integrated RIXS-MCD intensity for all m_j components for Co_2MnSi, as shown in Fig. 4 in the main paper, and for Mn_2VAl in Fig. S6.

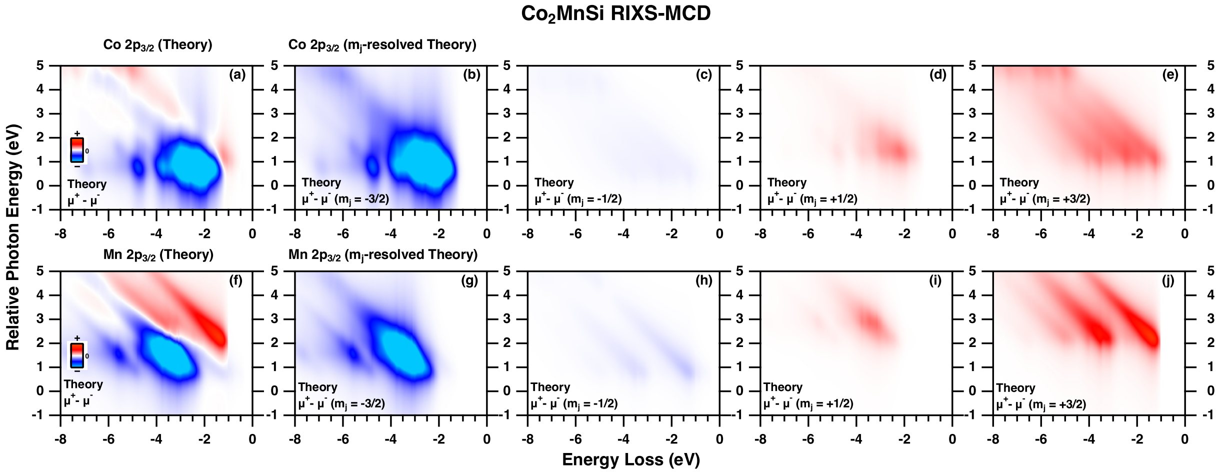

FIG. S4. Theoretical RIXS-MCD spectra for Co_2MnSi at the Co and Mn $2p_{3/2}$ edge in (a) and (f). The deconvoluted components into $m_j = -3/2, -1/2, +1/2$ and $+3/2$ states are shown in (b)(c)(d)(e) ((g)(h)(i)(j)) for the Co (Mn) edges.

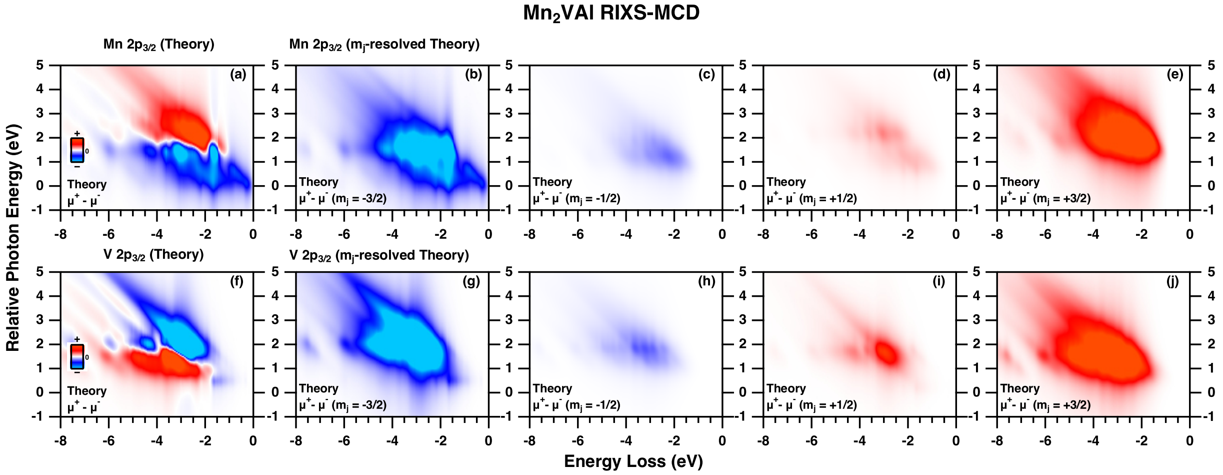

FIG. S5. Theoretical RIXS-MCD spectra for Mn_2VAl at the Mn and V $2p_{3/2}$ edge in (a) and (f). The deconvoluted components into $m_j = -3/2, -1/2, +1/2$ and $+3/2$ states are shown in (b)(c)(d)(e) ((g)(h)(i)(j)) for the Mn (V) edges.

In ferrimagneitc Mn_2VAl, the antiparallel coupling of the spin for Mn and V site gives the opposite order of the core-level Zeeman splitting for Mn and V $2p_{3/2}$ states, which is indicated by the vertical bars located at the MCD peak for $m_j = \pm 3/2$ components in Fig. S6. Therefore, the resonance energy of the $m_j = +3/2$ components in the V $2p_{3/2}$ edge is

lower than that of the $m_j = -3/2$ components, and the MCD contrast is reversed as shown in Fig. S5 ((f)(g)(j) compared with (a)(b)(e)), which is consistent with experimental data as shown in Fig. 3(e) compared with Fig. 3(f) in the main paper. Note that the halfmetallic gap of Mn_2VAl opens in the up-spin side as shown in the Fig. S7, and thus the gap of the $m_j = +3/2$ components in the V $2p_{3/2}$ edge (Fig. S5(j)) reflects the up-spin band gap of the V $3d$ PDOS as illustrated in Fig. S8(b). Since the core-level Zeeman splitting of 0.22 eV for the V $2p_{3/2}$ states is smaller than that for the Mn of 0.5 eV in Mn_2VAl [16], it is rather difficult to separately observe the $m_j = \pm 3/2$ components in the V $2p_{3/2}$ edge of Mn_2VAl as shown in Fig. S5(f). Although the precise estimation of the gap size could be difficult in this case, the positive MCD signals around 512.5 eV in the experimental V $2p_{3/2}$ RIXS-MCD spectra of Mn_2VAl (in Fig. 3(e) in the main paper) suggest the up-spin gap for the V $3d$ states.

On the other hand, the simulated RIXS-MCD spectra of the Mn edge for Mn_2VAl starts from 0 eV in the energy loss axis (Fig. S5(a) and (b)), which is consistent with the experimental RIXS-MCD spectra as shown in Fig. 3(f) in the main paper. Note that the Mn $3d$ states are dominant in the DOS around the E_F in the down-spin side as shown in the Fig. S7. This attributes to the negative MCD signals branching off from the elastic line (Fig. 3(f)) due to the spin polarized electronic structures around the Fermi level as illustrated in Fig. S8(a) [16].

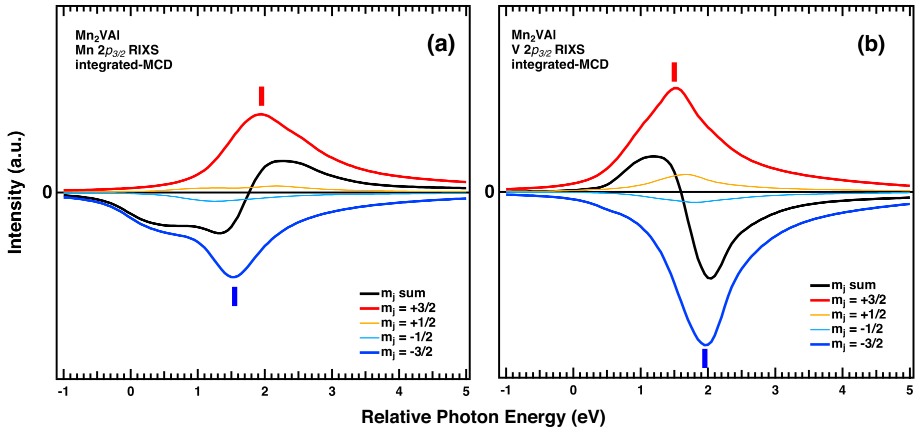

FIG. S6. Photon energy dependence of the simulation-derived RIXS-MCD intensity of Mn_2VAl at (a) Mn and (b) V $2p_{3/2}$ edges with the $m_j = -3/2, -1/2, +1/2$ and $+3/2$ components together with their sum.

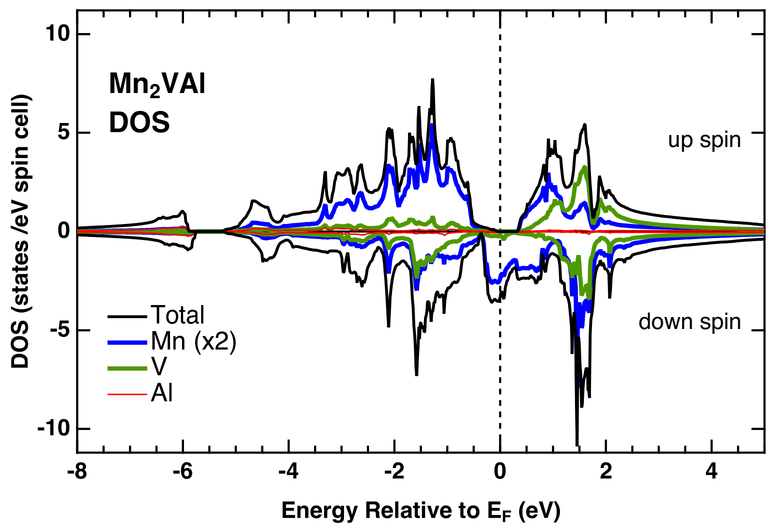

FIG. S7. The theoretical prediction of the spin-dependent DOS of Mn_2VAl and PDOS of Mn, V, and Al.

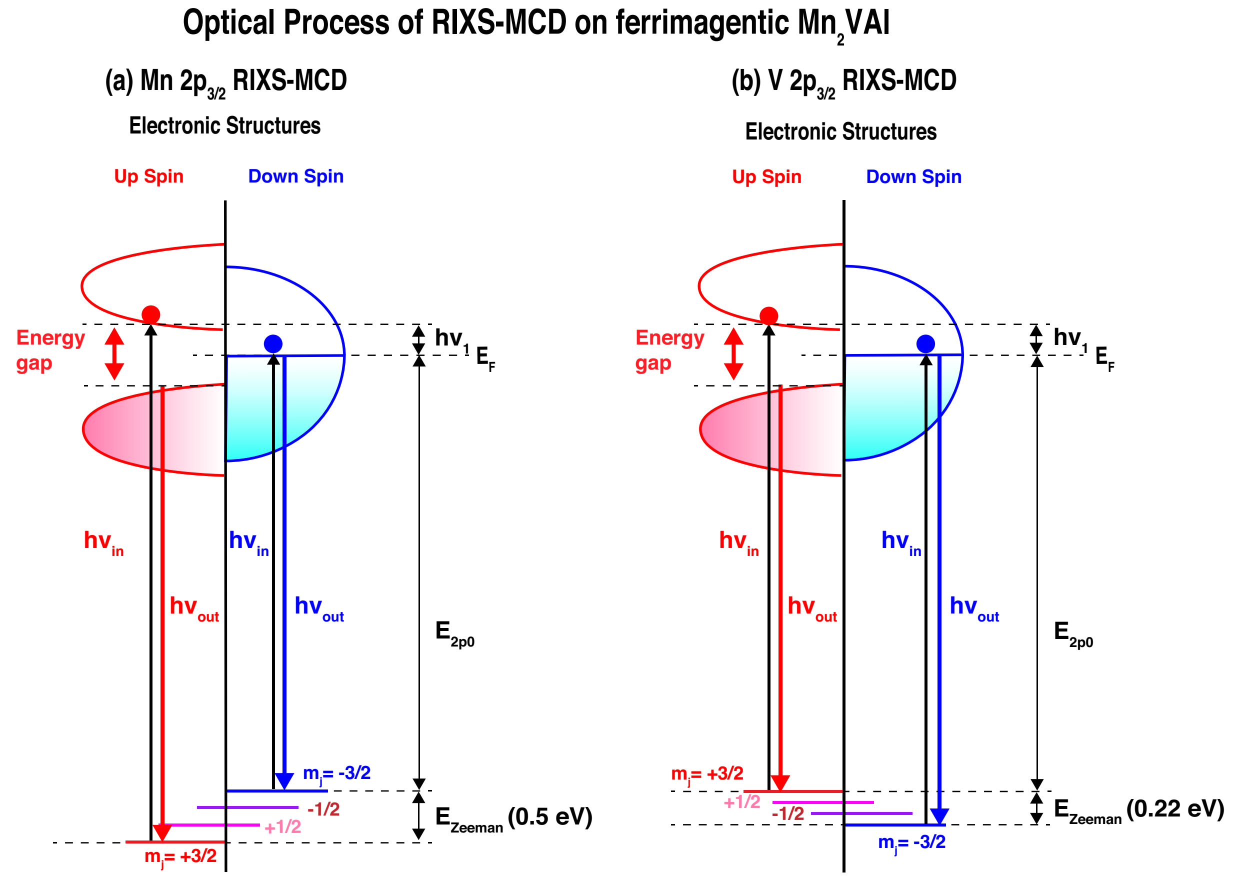

FIG. S8. The optical process of Mn RIXS-MCD on Mn_2VAl at (a) Mn and (b) V $2p_{3/2}$ edges

ESTIMATION OF THE DOWN-SPIN GAP IN THE RIXS-MCD

Figure S9(a) and (b) show the Co and Mn $2p_{3/2}$ RIXS-MCD spectra of Co_2MnSi obtained at the $m_j = \pm 3/2$ resonance, respectively. In the $m_j = -3/2$ spectrum, the down-spin gap ($E_{g_\downarrow}$) is estimated by the energy difference between the elastic peak and the onset energy of the RIXS-MCD signals. The onset energies of the RIXS-MCD spectra at the $m_j = -3/2$ resonance for the Co and Mn $2p_{3/2}$ edges are extracted by linear extrapolation as indicated by the green line in Fig. S9(a) and (b), respectively. Then, we obtained the $E_{g_\downarrow}$ for the Co $3d$ of 0.9 eV and that for the Mn $3d$ states of 1.9 eV.

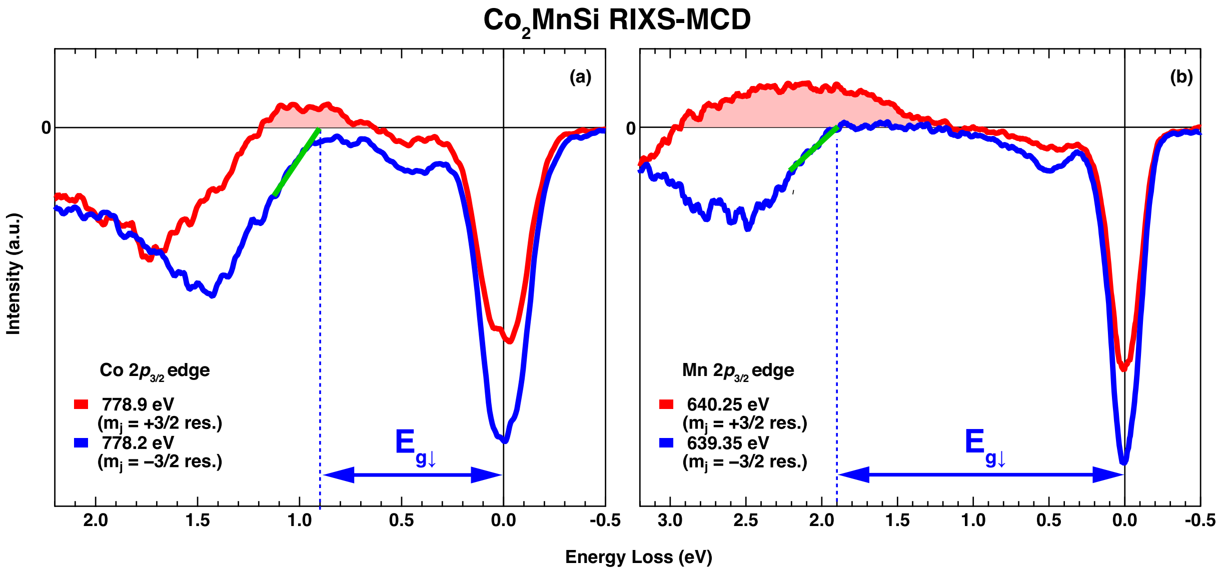

FIG. S9. RIXS-MCD spectra of Co_2MnSi recorded at the $m_j = \pm 3/2$ resonance for the (a) Co and (b) Mn $2p_{3/2}$ edges. The RIXS-MCD spectra are scaled to discuss the line shape. The solid green lines are guides to the eye to estimate the onset energies in the $m_j = -3/2$ spectra.

OFF-STOICHOMETRIC EFFECTS

It is well known that the off-stoichometric effects often modify the magnetic properties and the local electronic structure of the Heusler alloys [17]. To check the off-stoichiometry effects in Co_2MnSi, we have numerically simulated the electronic structures by using the Korringa-Kohn-Rostoker (KKR) method incorporated with the coherent-potential approximation (CPA) in the local density approximation (LDA) implemented by Machikaneyama-

2002 package [18] as demonstrated in Fig. S10. The atomic positions and the lattice constant were set to the same values described in the Method section in the main paper. Assuming that the Si sites are substituted by excess ions with 4% of Mn, which is evaluated by EPMA for the measured specimen, we obtained the total magnetic moment of 5.10 μ_B/f.u., which is comparable to that of the stoichiometric composition of 4.98 μ_B/f.u. in the simulation. Note that the total density of states are not significantly modified due to the off-stoichiometry effects within the simulation as shown in Fig. S10.

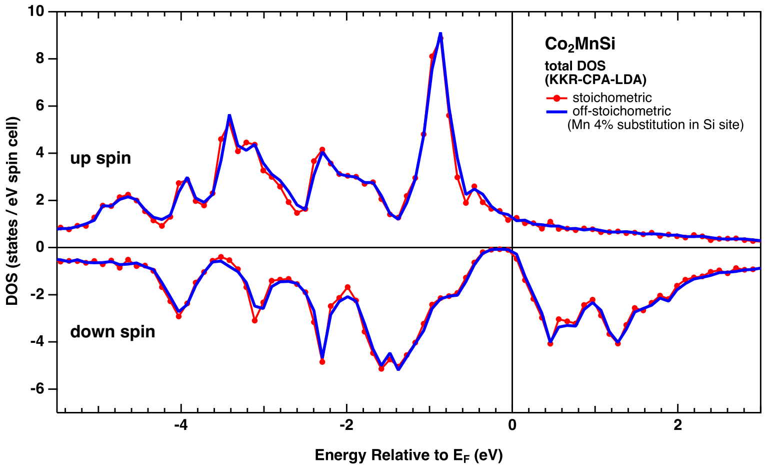

FIG. S10. Off-stoichiometric effects on total density of states of Co_2MnSi obtained by the KKR-CPA-LDA method.

* fujiwara@mp.es.osaka-u.ac.jp

[1] Saitoh, Y., et al., *J. Synchrotron Rad.* **19**, 388 (2012).

[2] Telling, N. D. et al., *Phys. Rev. B* **78**, 184438 (2008).

[3] Thole, B. T., Carra, P., Sette, F. & van der Laan, G., *Phys. Rev. Lett.* **68**, 1943 (1992).

[4] Carra, P., Thole, B. T., Altarelli, M. & Wang, X., *Phys. Rev. Lett.* **70**, 694 (1993).

[5] van der Laan, G. & Figueroa, A. I., *Coord. Chem. Rev.* **277**, 95 (2014).

[6] Goering, E., *Phil. Mag.***85**, 2895 (2005).

[7] Dürr, H. A., et al., *Phys. Rev. B* **56**, 8156 (1997).

[8] Edmonds, K. W., et al., *Phys. Rev. B* **71**, 064418 (2005).

[9] Nagai, K., et al., *Phys. Rev. B* **97**, 035143 (2018).

[10] Fujiwara, H. et al., *J. Synchrotron Rad.* **23**, 735 (2016).

[11] Sekiyama, A., et al., *New J. Phys.* **12**, 043045 (2010).

[12] Weinen, J., et al., *J. Electron Spectrosc. Relat. Phenom.* **198**, 6 (2015).

[13] Trzhaskovskaya, M. B., Nefedov, V. I. & Yarzhemsky, V. G., *At. Data Nucl. Data Tables* **77**, 97 (2001).

[14] Trzhaskovskaya, M. B., Nefedov, V. I., & Yarzhemsky, V. G., *At. Data Nucl. Data Tables* **82**, 257 (2002).

[15] Trzhaskovskaya, M. B., Nikulin, V. K., Nefedov, V. I., & Yarzhemsky, V. G., *At. Data Nucl. Data Tables* **92**, 245 (2006).

[16] Umetsu, R. Y., et al., *Phys. Rev. B* **99**, 134414 (2019).

[17] Tsunekawa, M. et al., *Jpn. J. Appl. Phys.* **54**, 082401 (2015).

[18] Akai, H. *J. Phys.: Condens. Matter* **1**, 8045 (1989).
